# Evaluation of *Cedrus atlantica* Essential Oil: Chemical Composition, Anticancer Activity and Molecular Docking Studies

**DOI:** 10.3390/molecules31010046

**Published:** 2025-12-22

**Authors:** Silvia Gruin, Octavian Crețu, Alexandra Mioc, Marius Mioc, Alexandra Prodea, Elisabeta Atyim, Alexandra Teodora Lukinich-Gruia, Maria-Alexandra Pricop, Armand Gogulescu, Codruța Șoica

**Affiliations:** 1 Faculty of Medicine, “Victor Babes” University of Medicine and Pharmacy, Eftimie Murgu Square, No. 2, 300041 Timișoara, Romania; silvia.gruin@gmail.com (S.G.);; 2 Faculty of Pharmacy, “Victor Babes” University of Medicine and Pharmacy, Eftimie Murgu Square, No. 2, 300041 Timișoara, Romaniaalexandra.ulici@umft.ro (A.P.); codrutasoica@umft.ro (C.Ș.); 3 Research Center for Experimental Pharmacology and Drug Design (X-Pharm Design), “Victor Babes” University of Medicine and Pharmacy, Eftimie Murgu Square, No. 2, 300041 Timișoara, Romania; 4 OncoGen Centre, Clinical County Hospital “Pius Branzeu”, Blvd. Liviu Rebreanu 156, 300723 Timișoara, Romania; alexandra.gruia@hosptm.ro (A.T.L.-G.); alexandra.pricop@oncogen.ro (M.-A.P.); 5 Faculty of Industrial Chemistry, Biotechnology and Environmental Engineering, Polytechnic University of Timisoara, Vasile Pârvan 6, 300223 Timișoara, Romania

**Keywords:** *Cedrus atlantica* essential oil, himachalane, cytotoxicity, natural products, anticancer activity, PEG-400 formulation, tween 20 formulation

## Abstract

Due to their high content of bioactive compounds with anticancer properties, essential oils (EO) are increasingly viewed as valuable therapeutic strategies in oncology. The aim of this study was to evaluate the chemical composition and anticancer activity of *Cedrus atlantica* EO (CAEO) and its PEG-400 and Tween 20 formulations. The gas-chromatography (GC) analysis revealed a sesquiterpene-rich profile, with β-himachalene (39.32%) as the major constituent, followed by α-Himachalene (16.76%) and γ-Himachalene (12.92%). The cytotoxicity studies, performed using Alamar Blue assay on normal HaCaT human keratinocytes and A375 human melanoma and HT-29 colorectal carcinoma cell lines, revealed that CAEO displayed minimal toxicity on HaCaT cells, while significantly reducing A735 and HT-29 cell viability, at any of the concentrations tested. The PEG- and Tween-based formulations of CAEO exhibited the same effect on cell viability as the simple water dispersion of CAEO. The immunofluorescence-based examination of cellular morphology suggested that CAEO induces apoptosis in both cancer cell lines: A375 and HT-29; this apoptosis-related mechanism was further supported by the caspase-3/7 assay, which revealed a significant increase in caspase-3/7 activity after CAEO treatment. To further investigate the underlying mechanism, the JC-1 staining and high-resolution respirometry assays demonstrated that CAEO induces mitochondrial membrane depolarization and reduced mitochondrial active respiration (OXPHOS). Molecular docking further indicated that isoledene and β-himachalene exhibit the highest predicted affinity for PI3Kγ, suggesting a potential involvement of PI3K-related signaling in the pro-apoptotic activity of CAEO. Together, these results suggest that CAEO induces apoptosis through a mitochondria-mediated mechanism.

## 1. Introduction

Cancer remains one of the most important global health challenges, accounting for nearly 10 million deaths every year [1]. Its impact transcends national boundaries since it affects both high-income and low- to middle-income countries. The increasing cancer burden can be attributed to aging populations, environmental factors, and lifestyle changes [2]. Moreover, cancer inflicts a substantial global economic impact not only through the direct costs of treatment and care but also through the long-term disability of the patient [3]. Therefore, anticancer research is not only a medical imperative but also a socio-economic priority.

While progress in chemotherapy, radiotherapy, immunotherapy, and targeted therapies have improved the outcome for many types of cancer [4], in many patients limited treatment options continue to exist, particularly against aggressive or drug-resistant tumors [5]. Additionally, the adverse effects associated with conventional treatments significantly diminish the quality of life in affected patients [6]. Therefore, there is an urgent need for more effective and less toxic cancer therapies.

Natural compounds have emerged as promising options in anticancer therapy due to their diverse biological activities and structural variety. Numerous plant-derived compounds, such as paclitaxel, vincristine, and camptothecin, have already been successfully introduced in clinical practice [7]. These bioactive molecules often exert their effects through multiple mechanisms, including the induction of apoptosis, inhibition of angiogenesis, and modulation of cellular signaling pathways [8]. Moreover, natural compounds may act synergistically with conventional therapies, potentially enhancing their efficacy and reducing their side effects [9].

Essential oils (EOs) are complex mixtures of volatile compounds derived from plants and have attracted an increasing scientific interest for their potential anticancer properties [10]. Several studies reported that certain EOs and their components, such as monoterpenes, sesquiterpenes, and phenolic compounds, can exert cytotoxic effects against various cancer cells by inducing apoptosis, inhibiting cell proliferation, and modulating oncogenic signaling pathways [11]. However, the current knowledge remains limited since most studies are confined to in vitro assays or in vivo tests using animal models. The EO derived from various *Cedrus* species have been valued for a very long time for their therapeutic effects on genitourinary, respiratory, musculoskeletal, dermatological, and various other inflammatory diseases [12,13]. Despite its therapeutic properties and long use in traditional medicine, EO from *Cedrus* species continues to be insufficiently investigated in present pharmacology. The variability of the EO composition related to the botanical source, the extraction method, or the storage conditions further complicates the translation to clinical use that needs reproducible, standardized extracts [14,15]. Compared to the EO extracted from other *Cedrus* species, such as *Cedrus deodara* and *Cedrus funebris*, which are better characterized for their anti-inflammatory or antimicrobial properties, *Cedrus atlantica* (Atlas cedar) EO (CAEO) has proved strong cytotoxic effects against cancer cell lines [16].

CAEO is of particular interest in anticancer research and due to its high content of sesquiterpenes such as himachalene, atlantone, and cedrol, which have been associated with cytotoxic and pro-apoptotic effects in cancer cells [17,18]. In vitro studies have reported the cytotoxic effects of the CAEO against various cancer cell lines, including liver [19], breast [17], and colon [20] cancers, mainly through the induction of oxidative stress, mitochondrial dysfunction, and apoptotic signaling [20,21]. Nevertheless, despite evidence of cytotoxic effects in various cancer models, the precise molecular mechanisms and targets of the CAEO constituents remain poorly identified, therefore underlining the need for further studies in order to fully assess its therapeutic potential.

In addition to its cytotoxic effects, CAEO has been investigated as adjuvant in combination with conventional chemotherapy due to its anti-inflammatory and antioxidant properties [22] that are able to reduce the side effects of conventional drugs [23]. However, although such applications are promising, they remain preliminary approaches thus making the scientific understanding of CAEO anticancer potential still incomplete; further research efforts are needed in order to consider CAEO a viable therapeutic option. In particular, there is limited information on CAEO effects on mitochondrial function, oxidative phosphorylation, and apoptosis in human melanoma and colorectal carcinoma cells. Furthermore, the contribution of individual CAEO components to potential molecular targets such as PI3K isoforms has not been explored.

To address these gaps, the current paper investigates the chemical composition of CAEO through GC-MS analysis as well as its biological activity in vitro against two cancer cell lines, A375 human melanoma and HT29 colorectal carcinoma; this studies were followed by in-depth investigation of its anticancer molecular mechanisms. The affinity of CAEO components toward several molecular targets was assessed by means of molecular docking.

## 2. Results

### 2.1. Chemical Composition of CAEO

The hydrodistillation of ground dried wood yielded 1.7% (wt) of a clear yellowish oil. GC-MS analysis identified the presence of three main components: α-Himachalene (16.84%), γ-Himachalene (13.00%), and β-Himachalene (39.23%), along with several other minor compounds. The chemical constituents of CAEO are depicted in Table 1.

### 2.2. DPPH-Based Antioxidant Activity of CAEO

The DPPH assay was used to assess the antioxidant activity of CAEO, with ascorbic acid serving as a positive control. The IC_50_ values were calculated using a logarithmic trendline. The results, including IC_50_ and the maximum radical scavenging activity (RSA) (%) observed, are presented in Table 2. RSA (%) for both CAEO and ascorbic acid showed a dose-dependent increase in antioxidant activity within the tested ranges; however, CAEO did not exceed the antioxidant potency of ascorbic acid.

### 2.3. CAEO Induced a Selective Cytotoxic Effect on A375 and HT-29 Cell Lines

**Figure 1:**
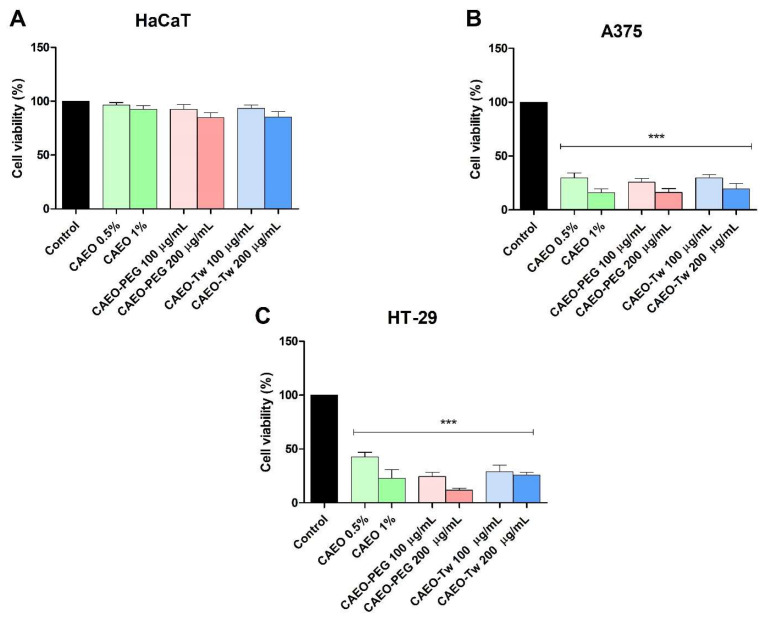
CAEO effect on HaCaT (**A**), A375 (**B**), and HT-29 (**C**) cell viability after 48h treatment with CAEO (0.5% and 1%), CAEO-PEG (100 and 200 μg/mL) and CAEO-Tw (100 and 200 μg/mL). Results are presented as viability percentages vs. control group (considered to be 100%). The data represent the mean values ± SD of three independent experiments performed in triplicate. The differences were statistically significant if *p* < 0.05 (*** *p* < 0.001).

The Alamar Blue assay was used to assess the viability of HaCaT, A375, and HT-29 cells following a 48 h exposure to varying concentrations of CAEO (0.5% and 1%), as well as CAEO formulated with PEG-400 (CAEO-PEG at 100 and 200 μg/mL) or Tween 20 (CAEO-Tw at 100 and 200 μg/mL). At any concentration, neither the simple water dispersion of CAEO, nor CAEO prepared with Tween 20 or PEG-400, resulted in a significant decrease in HaCaT cell viability when compared to the control group (100%) (Figure 1A).

The viability of A375 cell line following the same treatment with CAEO, CAEO-PEG, and CAEO-Tw, showed significant differences compared to the control. The following data demonstrated the significant differences in cell survival rates between the tested cells and the control group (100%): 29.63% ± 8.23 (CAEO 0.5%), 15.89 ± 6.40 (CAEO 1%), 25.81 ± 5.79 (CAEO-PEG 100 μg/mL), 16.19 ± 6.36 (CAEO-PEG 200 μg/mL), 29.66 ± 5.55 (CAEO-Tw 100 μg/mL) and 19.54 ± 8.95 (CAEO-Tw 200 μg/mL) (Figure 1B).

In HT-29 colorectal carcinoma cells, treatment with 0.5% and 1% CAEO reduced cell viability to 42.58% ± 7.64 and 22.64% ± 14.08, respectively, vs. control (100%). CAEO exhibited a more pronounced inhibitory effect when it was formulated with PEG-400 or Tween 20, reducing viability to 24.41% ± 7.05 (CAEO-PEG 100 μg/mL), 11.77% ± 2.83 (CAEO-PEG 200 μg/mL), 28.96% ± 10.71 (CAEO-Tw 100 μg/mL), and 25.79% ± 4.65 (CAEO-Tw 200 μg/mL) (Figure 1C).

### 2.4. CAEO Altered Nuclear and Cytoskeletal Appearance of A375 and HT-29 Cells

**Figure 2:**
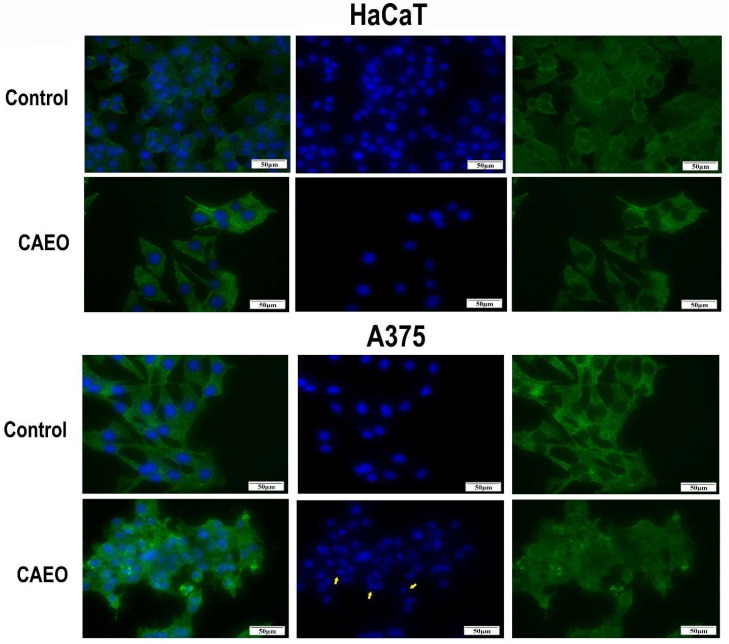

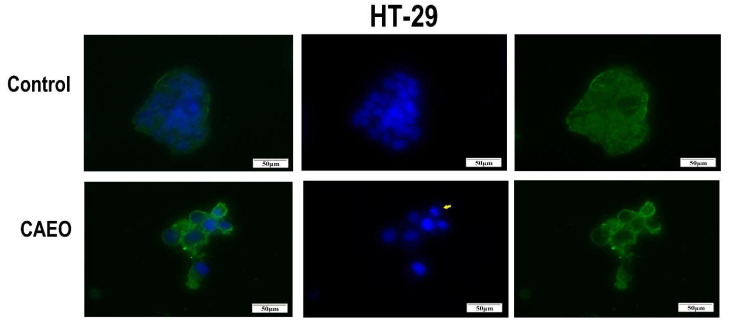
Immunofluorescence analysis of A375 and HT-29 cells treated with 1% CAEO. Blue staining represents cell nuclei, while the green staining represents beta-actin. The yellow arrows indicate signs of apoptosis. The scale bar is 50 μm.

The immunofluorescence analysis revealed that treatment with 1% CAEO induced morphological alterations of A375 and HT-29 cells, such as nuclear condensation/fragmentation and cytoskeletal collapse—hallmarks of apoptosis; normal HaCaT cells were unaffected (Figure 2).

### 2.5. CAEO Increased Caspase 3/7 Activity

**Figure 3:**
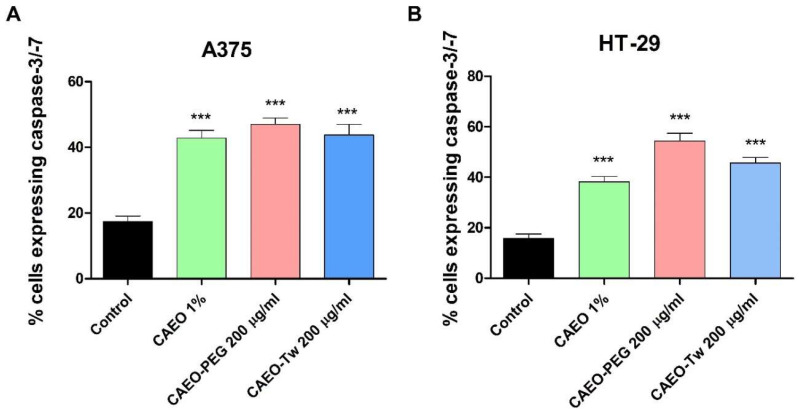
Caspase 3/7 activation in A375 (**A**) and HT-29 (**B**) cell lines after 48h treatment with CAEO 1%, CAEO-PEG and CAEO-Tw (200 μg/mL). The results are expressed as mean ± SD of three independent experiments. The differences were statistically significant if *p* < 0.05 (*** *p* < 0.001).

A crucial marker of apoptosis, caspase-3/7 activity, was measured in A375 and HT-29 cell lines following 48 h treatment with to CAEO 1%, CAEO-PEG (200 μg/mL) and CAEO-Tw (200 μg/mL). The findings, presented in Figure 3, revealed that treatment of both A375 and HT-29 cancer cells with a simple water dispersion of CAEO, as well as with CAEO formulated with PEG-400 and Tween 20, resulted in a significant increase in caspase-3/7 activity vs. control. The highest increase in caspase-3/7 activity occurred after treatment with 200 μg/mL CAEO-PEG for both cell lines, as follows: in the A375 cell line, 46.91 ± 3.4 vs. 17.43 ± 2.7 (control), and in HT-29, 54.30 ± 3.4 vs. 15.72 ± 3.1 (control) (Figure 3).

### 2.6. CAEO Influenced Mitochondrial Respiration and Mitochondrial Membrane Potential

**Figure 4:**
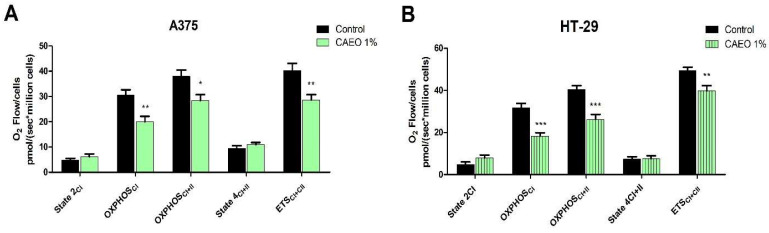
Mitochondrial respiratory parameters of permeabilized A375 (**A**) and HT-29 (**B**) cells after the 48h treatment with OEO 1%. The results are expressed as mean ± SD of three independent experiments. The differences were statistically significant if *p* < 0.05 (* *p* < 0.05, ** *p* < 0.01, *** *p* < 0.001).

As revealed by high-resolution respirometry studies, treatment of A375 and HT-29 cells with 1% CAEO induced notable changes in mitochondrial respiratory parameters (Figure 4). CAEO treatment decreased OXPHOS_CI_, OXPHOS_CI+II_ and ETS_CI+II_ respiratory rates in both A375 and HT-29 cell lines. Specifically, CAEO 1% lowered CI and CI+II ADP-stimulated respiration (OXPHOS_CI_ and OXPHOS_CI+II_) from 30.65 ± 3.5 to 20.03 ± 3.7 and 37.98 ± 4.3 to 28.93 ± 3.9 in the A375 cell line and from 31.68 ± 3.7 to 18.35 ± 2.6 and 40.48 ± 3.1 to 26.20 ± 4.1 in the HT-29 cell line. In a similar trend, CAEO treatment reduced the electron transport system’s (ETS_CI+II_) maximum capacity in A375 and HT-29 cell lines from 40.21 ± 4.9 to 28.76 ± 3.75 (A375) and from 49.52 ± 2.6 to 39 ± 4.3 (HT-29) (Figure 4).

**Figure 5:**
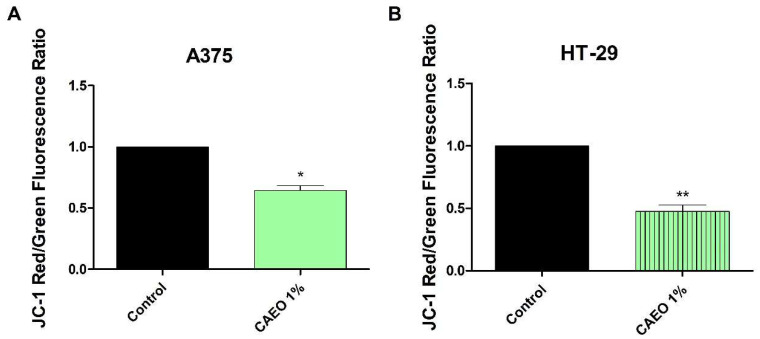
The JC-1 red/green fluorescence ratio of A375 (**A**) and HT-29 (**B**) cells treated with 1% CAEO. Values were normalized to the control group and represent mean ± SD of three independent experiments. The differences were statistically significant if *p* < 0.05 (* *p* < 0.05, ** *p* < 0.01).

The ΔΨm of A375 and HT-29 cells treated with 1% CAEO was evaluated using the fluorescent cationic dye JC-1. The results revealed a significant decrease in red/green fluorescence ratio vs. control (1), in both A375 (0.64 ± 0.06) and HT-29 (0.47 ± 0.09) cell lines; the significant decrease in fluorescence ratio in treated cells indicates that CAEO induces mitochondrial depolarization in both cancer cell lines (Figure 5).

### 2.7. Molecular Docking

Ligand-based molecular docking is a computational approach that can be used, among other applications, to begin understanding the targeted mechanism of action of a given molecular structure. The obtained data, in the form of free theoretical calculated binding energy values, may suggest an increased/decreased affinity of the examined molecule towards the specified target relative to the native ligand (a known inhibitor), given that calculated binding energy values decrease as compound theoretical affinity increases. In our current investigation, we employed a molecular docking-based protocol to discover potential protein targets for the 26 CAEO components, whose inhibition could relate to their in vitro anticancer cytotoxic effect. Protein targets, usually correlated with cancer development, elevated cell proliferation, and survivability within multiple types of cancer, such as epidermal growth factor receptor 1 (EGFR1), the vascular endothelial growth factor receptor 2 (VEGFR2), phosphoinositide-dependent kinase-1 (PDPK1), dual specificity mitogen-activated protein kinase 1 (MEK1), protein kinase B (AKT/PKB), phosphatidylinositol 4,5-bisphosphate 3-kinase catalytic subunits alpha and gamma isoforms (PI3Kα and PI3Kγ), mammalian target of rapamycin (mTOR), apoptosis regulator Bcl-2 (Bcl-2), and apoptosis regulator Bcl-XL (Bcl-XL), were used in the present work. Table 3 lists the docking scores (∆G, kcal/mol) for the 26 CAEO components and each protein’s native ligand used as a positive control.

**Figure 6:**
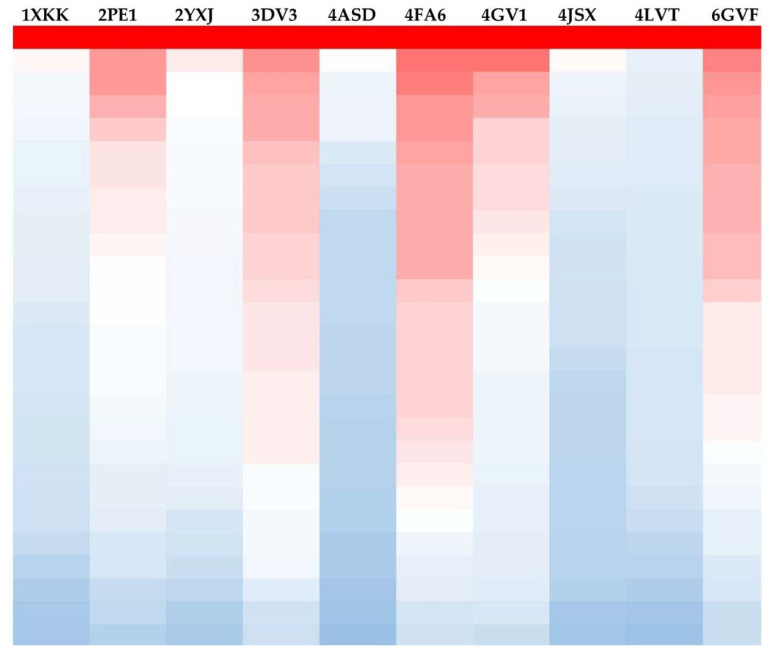
Heat map created based on Table 4 by coloring each reordered column in decreasing order using a three-color scheme (red-white-blue). Red represents the highest value (NL score), blue represents the lowest value, and white represents the midpoint interval.

None of the docked CAEO components outperformed the NLs of the protein targets. Nonetheless, to assess the collective impact of the 26 compounds on a single protein target, all gathered docking data were transformed into percentages of their corresponding NL’s docking result, with each NL receiving 100% (Table 4). Afterwards, a heatmap was created using Table 4 as a template. Each table column had its values rearranged in descending order, after which it was colored using a three-tone gradient: red for the maximum value (100%), white for the midpoint, and blue for values below 50%. Thus, the columns of the target proteins where most of the compounds obtained favorable docking scores relative to the native ligands will be colored mainly red (Figure 6). This visualization reflects relative docking trends and does not represent absolute binding affinities or true thermodynamic free energies.

**Figure 7:**
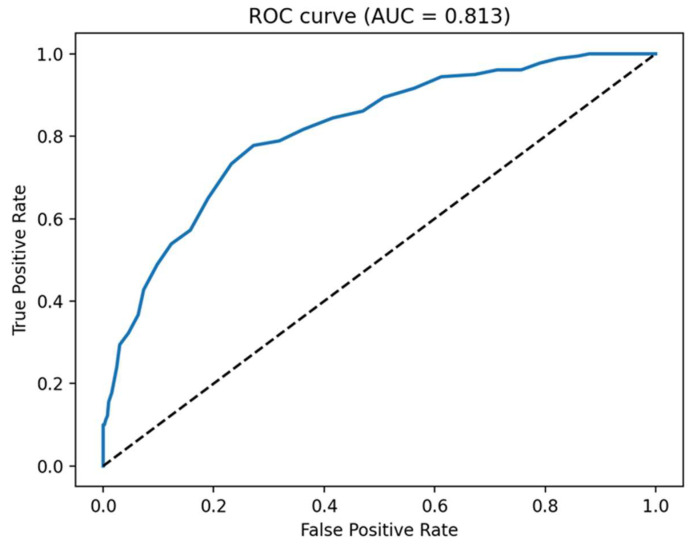
ROC curve evaluating the ability of the docking scoring function to discriminate PI3Kγ active ligands from DUD-E–generated decoys (AUC = 0.813). The dashed diagonal line represents random classification.

Based on the heatmap analysis, which indicated that PI3Kγ was the protein target toward which the majority of CAEO constituents exhibited the most favorable relative docking scores, this target was selected for further validation of the docking protocol. To evaluate the reliability of the applied scoring function, Receiver Operating Characteristic (ROC) analysis was therefore performed for PI3Kγ using experimentally reported inhibitors retrieved from the Protein Data Bank as active ligands and DUD-E–generated decoy molecules as non-binders. The resulting ROC curve yielded an area under the curve (AUC) value of 0.813, indicating good discriminatory performance of the docking protocol in distinguishing PI3Kγ-active ligands from decoys (Figure 7). This analysis supports the suitability of the docking protocol for comparative ligand ranking and target prioritization, while acknowledging that docking-based scores do not provide quantitative predictions of experimental binding free energies. Early recognition performance was further assessed using enrichment factor analysis. Given the moderate size of the validation dataset, EF was calculated at the 5% cutoff (EF 5%), yielding a value of 5.0 and indicating a five-fold enrichment of PI3Kγ-active ligands within the top 5% of the ranked list compared to random selection [24].

**Figure 8:**
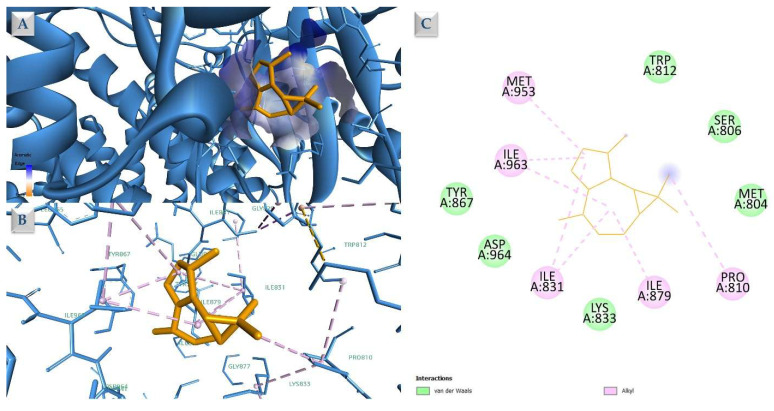
Compound **2** (Isoledene) docked into the PI3Kγ binding site (PDB ID: 4FA6). Shown are the slabbed 3D surface view (**A**), the full 3D representation (**B**), and the 2D map of ligand–protein interactions (**C**). Hydrophobic contacts are indicated by purple dashed lines.

**Figure 9:**
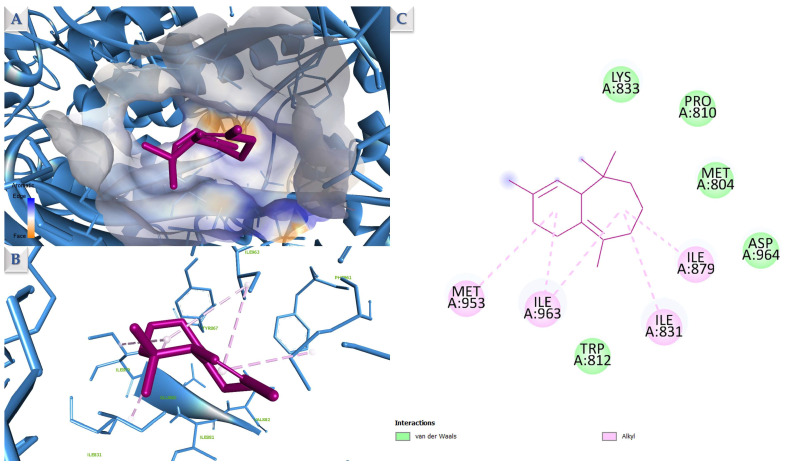
Compound **13** (β-Himachalene) docked into the PI3Kγ binding site (PDB ID: 4FA6). Shown are the slabbed 3D surface view (**A**), the full 3D representation (**B**), and the 2D map of ligand–protein interactions (**C**). Hydrophobic contacts are indicated by purple dashed lines.

The presented results show that most docked structures have a higher calculated affinity for PI3Kγ, followed by PI3Kα. In both protein score sets, compound **2** (Isoledene) is the most potent theoretical inhibitor, followed by the major compound **13** (β-Himachalene), which is ranked second in the docking list corresponding to PI3Kγ (4FA6). Given the preferential docking of CAEO constituents toward PI3Kγ and the validated discriminatory performance of the docking protocol, this target was selected for detailed interaction analysis of the top-ranked compounds. Being a sesquiterpene, Isoledene’s binding pattern involves mainly hydrophobic interaction formation with the neighboring amino acid residues present in the binding site of PI3K (Figure 8). These amino acids include: Pro810, Ile831, Ile879, Met953, and Ile963. Similarly, β-himachalene exhibits an overlapping interaction pattern, engaging ILE831, ILE879, ILE963, and MET953 through hydrophobic contacts (Figure 9). This interaction analysis shows that both Isoledene (compound 2) and β-himachalene (compound **13**) interact with the target by occupying a predominantly hydrophobic region of the PI3Kγ binding pocket. The residues contacted by both compounds are consistent with a lipophilic cavity within the PI3Kγ ATP-binding site that has been identified in structural and inhibitor design studies as a hydrophobic subpocket contributing to inhibitor binding and selectivity.

## 3. Discussion

Our study investigated the chemical composition and biological evaluation of CAEO to provide a preliminary insight into its potential anticancer activity. Gas chromatography revealed the presence of sesquiterpenes, himachalene isomers, as major components such as β-himachalene (39.23%), α-himachalene (16.84%), and γ-himachalene (13.00%); which totals of about 69% himachalene content, which is in good agreement with published *C. atlantica* wood oils and heartwood oils, where himachalenes typically account for around 60–70% of the composition and β-himachalene is the main constituent [25]. In contrast to several reported *C. atlantica* wood oils where oxygenated sesquiterpenes like atlantones and himachalol are significant constituents, our sample has comparatively higher proportions of δ-cadinene and other sesquiterpene hydrocarbons, indicating a hydrocarbon-rich chemotype and no major oxygenated derivatives among the identified constituents [26]

The DPPH assay revealed that CAEO possesses modest in vitro antioxidant activity compared with ascorbic acid (IC_50_ = 3704.83 µg/mL vs. 58.56 µg/mL). The lower efficacy can be attributed to its composition, which is rich in sesquiterpenes such as α-, ꞵ- and γ-himachalene, with inferior antioxidant activity compared to phenols like thymol, carvacrol and eugenol found in other EO known for their antioxidant effects [27,28]. Similar results were reported for the CAEO extracted from sawdust, which showed lower antioxidant activity compared to cedar wood tar EO or *Juniperus oxicedrus* EO [29]. *Juniperus oxicedrus* EO is rich in cedrol, a sesquiterpene that has reduced the oxidative stress in a mouse model of Alzheimer’s disease [30]. The superior antioxidant activity of *C. atlantica* wood tar EO compared to sawdust counterparts was also demonstrated by Jaouadi et al. [18], indicating that the extraction method directly impacts antioxidant activity. Therefore, while the CAEO tested may possess some innate antioxidant properties, its effect is inferior to that of vitamin C or other similar cedar EO, likely due to differences in origin and the extraction method used.

The current study aimed to evaluate the cytotoxic effects of CAEO as well as its PEG-400 and Tween 20-formulations against HaCaT human keratinocytes, A375 human melanoma, and HT-29 colorectal carcinoma cells. The obtained results challenge several previous literature reports in terms of bioactivity and cytotoxic selectivity as well as molecular mechanism. When tested in HaCaT cells, CAEO exhibited low cytotoxicity, with cell viability showing no significant decrease compared to the control. EO, including CAEO, have been reported in the literature as cell membrane disruptors due to their lipophilic nature which allows their incorporation into the hydrophobic regions of the membrane; such effects have been identified as mechanisms underlying their antibacterial activity [31] as well as their non-selective toxicity. Several hypotheses can be formulated to support such results; firstly, as revealed by gas chromatography, CAEO contains high amounts of sesquiterpenes such as β-himachalene, which exhibit lower volatility, slower diffusion, and presumably lower reactivity against mammalian cell membranes than monoterpenes [32,33], the latter being more commonly associated with cytotoxic activity through membrane-disruptive effects. Secondly, cell membrane differs significantly between bacteria and mammals [34]. Mammalian cells lack a peptidoglycan layer but benefit from robust detoxification pathways such as the cytochrome P450 system and antioxidants [35,36,37]; therefore, CAEO may exert selective toxicity against bacteria. More specifically, HaCaT cells are spontaneously immortalized keratinocytes characterized by high metabolic activity and resistance to stress, which made them less sensitive to external agents [38]. Such resilience can be related to the expression of membrane-stabilizing proteins such as E-cadherin or HuR [39] as well as other relevant proteins such as the Na+/K+-ATPase, claudins, and occludins [40].

Thirdly, CAEO components may present low bioavailability in HaCaT cells due to their large molecular size, causing poor diffusion, thus resulting in the absence of cytotoxicity. In fibroblasts, however, EO containing sesquiterpenes significantly increased cell membrane fluidity as well as cytotoxicity compared to monoterpenes [41]. Alternatively, normal keratinocytes display a slightly alkaline pH [42] and specific lipid composition that may limit the disruptive potential of lipophilic CAEO components, unlike malignant cells that often show altered membrane profiles [43].

We used two emulsifiers, PEG-400 and Tween 20, in order to produce the samples tested in a biological environment; some studies reported that such emulsifiers are able to reduce the cytotoxicity of EO by their encapsulation into micro- and nanoemulsions [44]. In our study, however, the presence of PEG-400 and Tween 20, respectively, increased the cytotoxic effects in malignant cells. The vesicular transport mediated by the two emulsifiers may facilitate the cell internalization of CAEO components through endocytosis, thus enhancing their delivery to mitochondria, where apoptosis occurs [45]. Additionally, both emulsifiers are able to act as cell membrane penetration enhancers, thus facilitating the entrance of CAEO components into the malignant cells [46,47]. Moreover, Tween 20 has been reported to inhibit drug efflux pumps like P-glycoprotein, thus retaining the active compounds inside the cancer cells [48].

In A375 and HT-29 cells, our study revealed a substantial reduction in cell viability following the application of the CAEO; in particular, CAEO induced very strong cytotoxic effects (as low as 11.77% viability with CAEO-PEG at 200 μg/mL) in HT-29 cells whose sensitivity suggests a targeted activity. So far, the literature reported very limited data regarding the cytotoxicity of CAEO in malignant cells; one study revealed a significant cytotoxic activity against MCF-7 breast cancer cells with an IC_50_ value of 143.13 ± 14.6 µg/mL [17]. Another study reported that β-2-himachalene-6-ol (A β-himachalene DERIVATIVE) extracted from *Daucus carota*, demonstrated a potent anticancer activity against skin melanoma B16F-10, astrocytoma Caco-2, breast MDA-MB-231, lung adenocarcinoma A549 and astrocytoma SF-268 cancer cell lines (IC_50_ 4–13 µg/mL; 18–58 µM), inducing cell death through apoptosis [49]. Indeed, the terpenoids contained in CAEO may induce apoptosis by directly affecting the mitochondrial integrity and releasing intrinsic apoptotic triggers such as caspases; in particular, HT-29 colon cells can be used as an excellent surrogate to study drugs that affect mitochondrial complexes [50].

Although CAEO exhibited only modest antioxidant activity in the DPPH assay, this finding suggests that its cytotoxic effects are unlikely to be directly attributable to radical scavenging capacity alone. This observation indicates that antioxidant activity does not play a dominant role in the biological effects observed in cancer cells, and that alternative, non-antioxidant mechanisms—such as those explored in subsequent sections—are likely to contribute to CAEO-induced cytotoxicity.

Given the apoptosis-related features observed by immunofluorescence and to validate this premise, we assessed the activation of a key hallmark of apoptosis, caspase-3/7. Our study revealed that CAEO significantly increased caspase-3/7 activity, thus indicating the activation of the apoptotic executory phase; while several studies have reported its cytotoxic activity against various cancer cell lines, few have investigated the underlying molecular mechanisms. As an example, one study revealed that CAEO induced moderate cytotoxicity against MCF-7 breast cancer cells but did not investigate whether this effect occurred through apoptosis or necrosis [17]. Huang et al. reported concentration- and time-dependent cytotoxic effects in two colorectal cancer cell lines, including HT-29, and although they identified certain underlying mechanisms, they did not assess the direct CAEO effects on caspases [20]. Our findings provide evidence that CAEO activates executioner caspases; this aligns with previous studies on hepatocellular carcinoma [19] and glioblastoma [21] cells where CAEO regulated p53/p21 and CDK4/cyclin D1 protein expression and induced extrinsic and intrinsic apoptosis.

After confirming an apoptotic response through caspase-3/7 activation and to clarify the underlying mechanism, the next aim of this study was to evaluate the involvement of mitochondria in the cytotoxic effect of CAEO on A375 and HT-29 cells. Our results revealed that CAEO decreased OXPHOS_CI_ and OXPHOS_CI+II_, thus indicating that CAEO inhibited oxidative phosphorylation and induced mitochondrial dysfunction. To the best of our knowledge, this is the first study to report the potential of CAEO to inhibit mitochondrial respiration in A375 and HT-29 cancer cells. Moreover, CAEO was able to induce mitochondrial membrane depolarization in A375 and HT-29 cancer cell lines. The decrease in ΔΨm and oxidative phosphorylation showed that CAEO is able to disrupt electron transport and compromise ATP synthesis. Loss of ΔΨm is a hallmark of intrinsic (mitochondria-dependent) mediated apoptosis, and such depolarization has been reported to precede cytochrome c release and caspase activation [51,52]. Taken together, our findings suggest that CAEO may exert its cytotoxic activity through mitochondrial dysfunction and activation of mitochondria-dependent cell death pathways. A similar cytotoxic mechanism, permeabilization of mitochondrial membrane and release of caspase that led to apoptosis, was described after the treatment of various cancer cells with numerous EO, or EO components [11,23]. The EO of another species belonging to the same genus, *Cedrus deodara*—containing high levels of β-himachalene and α-himachalene, has been reported to induce ΔΨm loss, caspase-3 activation and apoptosis in colon cancer cell lines, suggesting a similar mitochondrial involvement in its cytotoxic effect [53]. In our study, the presence of PEG-400 and Tween 20 increased the cytotoxic effects in malignant cells and caspase-3/7 activation; however, the mitochondrial assays (ΔΨm and mitochondrial respiration) were performed using the simple water dispersion of CAEO. Therefore, the observed mitochondrial effects are attributed to the activity of CAEO itself, independent of the emulsifiers. Nonetheless, the increase in the in vitro cytotoxic effects for the formulated CAEO highlights that delivery and pharmacokinetics could influence CAEO activity in vivo, and these effects warrant further investigation in future studies.

A critical consideration is the variability of CAEO samples in terms of chemical composition, which depends on the geographic origin, harvest time, and extraction method [54]. Sesquiterpenes presumably contribute to the recorded pro-apoptotic activity since other sesquiterpene compounds, such as β-caryophyllene, have already been identified as anti-carcinogenic agents through apoptosis induction, intervention in the cell cycle, and inhibition of proangiogenic, invasive and proliferative factors [55].

Molecular docking revealed that PI3Kγ was the most advantageous target for CAEO constituents, while Isoledene (compound **2**) and β-himachalene (compound **13**) demonstrated the strongest calculated affinities among all tested molecules. Although ROC analysis demonstrated good discriminatory performance of the docking protocol, such validation primarily reflects the ability to distinguish active ligands from decoys rather than to predict absolute binding affinities. Therefore, docking results should be interpreted as qualitative indicators of relative interaction propensity and used to support mechanistic hypotheses rather than to infer definitive inhibitory potency.

The obtained calculated scores of compounds **2** and **13** are in line with published biological data on other himachalene-type sesquiterpenes, such as β-2-himachalene-6-ol, which reduced p-Akt and p-Erk and increased apoptosis in models of melanoma and colon cancer. These sesquiterpenes have been shown to have anticancer activity linked to PI3K/AKT and MAPK pathway suppression [56,57]. Similar to the mitochondrial dysfunction and caspase-3/7 activation exhibited in A375 and HT-29 cells by our study, isoledene-rich plant fractions have been demonstrated to cause ROS generation, ΔΨm loss, cytochrome c release, and caspase-8/9/3 activation in HCT116 colon cancer cells [58]. All of these results suggest that the sesquiterpene-rich profile of CAEO may activate PI3K isoforms, especially PI3Kγ, as upstream regulators of the experimentally discovered mitochondrial apoptotic cascade. This establishes a logical mechanistic connection between the docking results and the biological effects that CAEO caused in cancerous cells.

Taken together, these findings suggest that CAEO, particularly when formulated with PEG-400 or Tween 20, exerts selective cytotoxicity against malignant cells through mechanisms that may involve mitochondrial damage and apoptosis induction. However, further mechanistic investigations, such as complementary ΔΨm assays (TMRE/TMRM), cytochrome c release, and apoptotic cascade analysis (Western blot for apoptotic proteins) and Mitochondrial permeability transition pore (MPTP) assays will be fundamental to validate and refine the mitochondrial mechanism proposed in this study.

## 4. Materials and Methods

### 4.1. EO Extraction and GC-MS Analysis

The dried wood (Voucher number VSNH.BUASTM-125) was kindly provided by “King Michael I” University of Life Sciences (Timisoara, Romania Herbarium). The dried plant material was crushed and steam distilled for 4 h at 100 °C in a Craveiro-type apparatus [59,60]. As previously described [61], steam was produced by heating a 3 L glass boiler with electrical resistance that had previously been filled with water. The water tank was refilled when necessary. The steam was then directed to the bottom of the 1 L glass extraction container. A water-cooling system was utilized to condense the steam after it had passed through the plant material, which was put on a perforated plate a few centimeters from the bottom of the extraction tank. Finally, to avoid artifacts caused by overheating, CAEO and hydrosol (aqueous phase) were collected in a 250 mL glass receiver equipped with a water-cooling jacket and a hydrosol overflow outlet. The oil was treated with anhydrous sodium sulfate, after separation, to eliminate water residues. It was then stored in sealed vials at −18 °C for future analysis. The extraction yield was estimated using the formula: oil weight/dry plant weight × 100 (wt).

The material was analyzed using a GC Hewlett Packard HP 6890 Series gas chromatograph and a Hewlett Packard 5973 Mass Selective Detector (Agilent Technologies, Inc., Santa Clara, CA, USA). A 1 μL diluted sample (1:100 in hexane) was injected into the gas chromatograph with the following set of parameters: 50 °C to 250 °C temperature range with a rate of 6 °C/min, DB-WAX capillary column (Agilent Technologies, Inc., Santa Clara, CA, USA) (30 m length, 0.25 mm internal diameter, 0.25 μm film thickness), and a 4 min solvent delay. The mass spectrometer was set to 230 °C, the MS Quad at 150 °C, and the helium gas flow was 1 mL/min. The masses of the investigated compounds ranged from 50 to 600. The resultant spectra were compared to data from the NIST 02 library (NIST Mass Spectral Library, Version 2.0, National Institute of Standards and Technology (NIST) Gaithersburg, MD, USA), and the area percentage was calculated. The retention indexes were calculated based on the retention times and areas of C9 to C18 alkanes.

### 4.2. DPPH-Based Antioxidant Activity

Reagents, 2,2-diphenyl-1-picrylhydrazyl (DPPH), ascorbic acid, and methanol were obtained from Merck (Merck KGaA, Darmstadt, Germany). The antioxidant activity was evaluated using the 2,2-diphenyl-1-picrylhydrazyl (DPPH) assay, as previously described [62]. This method is based on the reduction in the single-electron N atom of DPPH to the corresponding hydrazine in the presence of an H-donating antioxidant, leading to the discoloration of the initial violet solution and a subsequent decrease in absorbance at 517 nm [63]. Various dilutions of cedar EO (156.25–10,000 µg/mL) and ascorbic acid (25–200 µg/mL), used as a positive control, were prepared in methanol. Then, 0.2 mL of methanol (used as a blank), the cedar EO and ascorbic acid dilutions were added to 1.8 mL of DPPH 0.1 mM in methanol and incubated at room temperature for 30 min. Afterward, the absorbance was measured with a Shimadzu UV-1900i spectrophotometer (Shimadzu Scientific Instruments Inc., Columbia, MD, USA) at 517 nm. The radical scavenging activity (RSA) was calculated using the following formula:

(1) $$RSA(\%)=\;[\frac{Ao-As}{Ao}]\times \;100$$

where *Ao* = absorbance of the blank, *As* = absorbance of the sample

### 4.3. Cell Culture

The cells selected for our study were human keratinocytes HaCaT cells, acquired from CLS Cell Lines Service GmbH (Epplheim, Germany), A375 human melanoma cells and HT-29 colorectal adenocarcinoma cells, purchased from American type Culture Collection, ATCC (Lomianki, Poland). The cells were acquired as frozen items and stored in liquid nitrogen. HaCaT and A375 cells were cultured in Dulbecco’s Modified Eagle Medium high glucose, supplemented with 10% FBS and 1% penicillin/streptomycin, while HT-29 cells were propagated in McCoy’s 5A Medium (Thermo Fisher Scientific, Waltham, MA, USA), supplemented with 10% FBS and 1% antibiotic mixture. The cells were maintained in a humidified incubator at 37 °C with 5% CO_2_.

### 4.4. Cell Viability Assessment

Alamar blue. The cytotoxic activity of CAEO was assessed by means of Alamar blue assay (Thermo Fisher Scientific, Waltham, MA, USA). The cells (1 × 10^4^/well) were seeded onto 96-well plate and allowed to attach for 24h until reaching 80–85% confluence. The next day, the old medium was removed and replaced with fresh medium, specific for each cell line, containing the tested concentrations of CAEO dispersed in fresh media (0.5% and 1%) and CAEO formulated with PEG-400/Tween 20 (CAEO-PEG and CAEO-Tw 100 μg/mL, 200 μg/mL). After 48 h, 20 μL of Alamar blue reagent was added to each well and the plates were incubated for 3 h at 37 °C. The absorbance measurements were carried out at 570 nm using a microplate reader (xMark™ Microplate Spectrophotometer, Biorad, Hercules, CA, USA).

### 4.5. Immunofluorescence-Based Examination of Cellular Components

To evaluate the cellular morphology, immunofluorescence staining of β-actin and nuclei was performed on HaCaT, A375 and HT-29 cells. Briefly, the staining protocol was as follows: the cells (2 × 10^5^ cells/well) were seeded in 12-well plates, allowed to reach 80–85% confluence and treated for 48h with 1% CAEO. After treatment, the cells were washed with PBS, fixed 10 min with 4% paraformaldehyde, permeabilized for 15 min with 0.1% Triton X and blocked for 30 min with BSA (3%). Next, the cells were stained (1 h, room temperature) with beta-actin mouse monoclonal antibody (1:2000) in blocking buffer, and then incubated (30 min, room temperature, in the dark) with a secondary antibody, Alexa Fluor Plus 488 conjugate (1:500). Additionally, to evaluate the nuclear localization and morphological changes, the nuclei were stained (10 min, room temperature, in the dark) with Hoechst 33,342 (1:2000). All products used in this study were purchased from Thermo Fisher Scientific, Inc., Waltham, MA, USA.

### 4.6. Caspase 3/7 Activity

The CellEvent Caspase-3/7 Green Detection Reagent Kit (Thermo Fisher Scientific, Waltham, MA, USA) was used to measure CAEO’s ability to induce apoptosis. The Countess 3 FL Automated Cell Counter (Thermo Fisher Scientific, Waltham, MA, USA) was used to analyze the stained cells treated with CAEO 1%, CAEO-PEG, and CAEO-Tw (200 μg/mL) in accordance with the manufacturer’s instructions [64]. This detection technique uses a dye that conjugates to a DEVD peptide sequence unique to caspase-3/7 and stays inactive. The DEVD peptide is cleaved when caspase-3 or caspase-7 is activated during apoptosis, enabling the dye to attach to DNA and release green fluorescence (excitation/emission: 502/530 nm).

### 4.7. High-Resolution Respirometry

Using the Oxygraph-2k high-resolution respirometer (Oroboros Instruments, Innsbruck, Austria) and a modified substrate–uncoupler–inhibitor titration (SUIT) protocol, previously described by Petruș et al. [65], mitochondrial respiration was measured at 37 °C. A375 and HT-29 cells were cultured and treated with 1% CAEO for 48 h and then trypsinized and resuspended (1 × 10^6^ cells/mL) in Miro 5, a specific mitochondrial respiration buffer (KH_2_PO_4_ 3 mM, EGTA 0.5 mM, K-lactobionate 60 mM, D-sucrose 110 mM, HEPES 20 mM, taurine 20 mM, MgCl_2_ 10 mM and BSA 1 g/L, pH adjusted at 7.1) was used to cultivate, trypsinize, and resuspend A375 and HT-29 cells/mL). The cells were inserted in the chambers of the device and left to stabilize for 15 min, before adding digitonin, a substance used to permeabilize the cellular membrane. Sequential additions of glutamate and malate (complex I—CI substrates), ADP, and succinate (complex II—CII substrate), oligomycin (inhibitor of ATP synthase), FCCP (Carbonyl cyanide-p-trifluoromethoxyphenylhydrazone, a protonophore) and antimycin A (a complex III inhibitor) allowed the measuring of the following mitochondrial respiratory rates: State 2_CI_, OXPHOS_CI_, and OXPHOSC_I+II_, State 4_CI+II_ and ETS_CI+II_ and residual oxygen consumption (ROX); all rates were corrected after ROX.

### 4.8. JC-1 Mitochondrial Membrane Potential (ΔΨm) Assay

The fluorescent cationic carbocyanine dye—JC-1 was used to measure the mitochondrial membrane potential with the Countess 3 FL Automated Cell Counter (Thermo Fisher Scientific, Waltham, MA, USA). JC-1 has the ability to selectively enter healthy mitochondria, aggregate and emit red fluorescence; in damaged mitochondria, with low ΔΨm, JC-1 will remain in the cytoplasm and emit green fluorescence. Briefly, the protocol used was as follows: the cells were cultured, treated with 1% CAEO for 48 h, washed with PBS and stained with JC-1 (10 μM) and incubated for 30 min at 37 °C. The cells were then washed again with PBS and transferred into a Countess chamber slide. The fluorescence was detected using GFP 2.0 and RFP 2.0 light cubes (Thermo Fisher Scientific, Waltham, MA, USA).

### 4.9. Molecular Docking

Molecular docking studies were conducted following an established computational workflow. The protein structures used as docking targets were downloaded from the RCSB Protein Data Bank (PDB) [66] and are summarized in Table 5. Depending on the availability of structural data, either full-length proteins or crystallized functional fragments, such as kinase domains or BH3-interaction regions, were selected. Table 5 lists the corresponding residue intervals and the lengths of the protein segments incorporated in the docking experiments. Docking was performed on biologically relevant binding pockets. These sites were defined based on the spatial coordinates of co-crystallized ligands when present, or from literature reports describing the functional domains responsible for catalytic or regulatory activity. Key residues forming these active or binding sites were determined through inspection of the PDB structures as well as published structural analyses. Chemical structures of the EO constituents were obtained from PubChem in SDF format. Each compound was then converted into an energy-minimized, 3D docking-ready format with the Open Babel tool integrated in PyRx v0.8 [67]. The docking settings and grid parameters used for each protein target, as generated through the PyRx workflow, are provided in Table 5. To ensure the reliability of the docking protocol, the native ligands co-crystallized with each protein were retrieved and re-docked under identical conditions. These ligands served as positive controls because they represent experimentally validated binders for their respective targets.

### 4.10. ROC Analysis and EF Calculation

All data processing and statistical analysis were performed using Python 3.11 in a Jupyter-kernel computational environment. The following libraries were used: (i) pandas 1.5 for data handling; (ii) scikit-learn 1.3 for ROC and AUC computation; (iii) matplotlib 3.8 for figure generation.

Docking scores were generated independently using AutoDock Vina (version 1.1.2) through the PyRx GUI (version 0.8). Only the scoring output (“Binding Affinity”) was used in this analysis.

Ligand sets were composed of 20 known active molecules (with existent biological data), retrieved from RCSB Protein Data Bank (https://www.rcsb.org/) and 100 decoy ligands obtained by using the DUD-E database. SMILE strings for these compounds are available in Table S1 within the Supplementary Materials. The dataset (active + decoys) was imported from Excel spreadsheets (.xlsx format), containing ligand identifiers, Vina calculated binding affinities, and binary activity labels (1 = active, 0 = decoy).

Calculated binding affinity values were cleaned using automated preprocessing steps: removal of non-numeric characters (e.g., ‘kcal/mol’), whitespace trimming, normalization of decimal separators (comma to period), and conversion to numeric types. Rows containing invalid or missing affinity values were discarded.

Because AutoDock Vina assigns more negative scores to stronger binders, affinity values were sign-inverted prior to ROC analysis:

score_adj = −Affinity

ROC curve was computed using scikit-learn’s roc_curve function, and AUC values were calculated with the auc function. This yields the standard, publication-ready ROC–AUC metrics used throughout cheminformatics and virtual screening research.

Early recognition performance was further evaluated using enrichment factor (EF) analysis. EF was calculated according to the following formula:

$${EF}_{x\%}=\frac{a/n}{A/N}$$

where *N* represents the total number of molecules in the validation dataset, *A* is the total number of active ligands, *n* corresponds to the number of top-ranked molecules within the selected cutoff x%, and *a* is the number of active ligands found within this top-ranked subset. Given the moderate size of the validation dataset, EF was calculated at the 5% cutoff (EF 5%)

### 4.11. Statistical Analysis

One-way ANOVA followed by Dunnett’s post-test was used for statistical analysis (GraphPad Prism 6.0, San Diego, CA, USA). If *p* < 0.05 (* *p* < 0.05, ** *p* < 0.01, and *** *p* < 0.001), the differences between the experimental groups were considered to be statistically significant.

## 5. Conclusions

In this study, we showed that CAEO extracted from steam-distilled wood exhibits a chemical profile dominated by β-, α-, and γ-himachalene. This composition indicates a hydrocarbon-focused profile with few oxygenated derivatives and is in line with previously reported chemotypes. The in vitro studies showed that CAEO had a selective cytotoxicity toward A375 human melanoma and HT-29 colorectal carcinoma cells while sparing normal HaCaT keratinocytes, suggesting a promising anticancer activity; however, further in vivo studies and pharmacological evaluation are necessary to substantiate its therapeutic potential. Nuclear condensation, caspase-3/7 activation, loss of mitochondrial membrane potential, and inhibition of oxidative phosphorylation all supported the induction of apoptosis, suggesting a mitochondria-dependent apoptotic mechanism. Molecular docking further showed that several CAEO constituents, especially isoledene and β-himachalene, show preferential affinity for PI3Kγ and PI3Kα, indicating that modulation of PI3K-related signaling may contribute to the observed biological effects. When considered collectively, these results suggest that CAEO is a promising natural product candidate for anticancer treatment. Its activity seems to result from a combination of interference with oncogenic kinase pathways and mitochondrial dysfunction. To completely determine CAEO’s therapeutic potential, future research should include in vivo evaluation, quantification of molecular targets, and thorough mechanistic validation.

## Figures and Tables

**Table 1:** Chemical composition of CAEO determined by GC–MS.

No.	Compound Name	RT	RI Calc	Area % Calc	Qual%
**1**	Ylangene	11.58	1290	0.067	>90
**2**	Isoledene	12.93	1361	1.15	>90
**3**	Limona ketone	13.00	1365	0.51	>90
**4**	Juniperol	13.31	1381	0.87	>90
**5**	1-Methyl-4-(1-methyl-2-propenyl)benzene	13.54	1393	1.30	>90
**6**	Cedrene	13.87	1411	0.59	>90
**7**	Himachala-2,4-diene	13.98	1417	0.51	>90
**8**	Thujopsene	14.33	1435	0.08	>90
**9**	α-Himachalene	14.79	1459	16.84	>90
**10**	β-bisabolene	15.13	1477	0.25	>90
**11**	α-Longipinene	15.46	1495	0.38	>90
**12**	γ-Himachalene	15.73	1509	13.00	>90
**13**	β-Himachalene	16.12	1530	39.23	>90
**14**	Calamene trans	16.22	1535	0.19	>90
**15**	1,1,4a-trimethyl-5,6-dimethylenedecahydronaphthalene	16.35	1541	0.63	>90
**16**	Isolongifolene,9,10-dehydro-	16.68	1559	0.42	>90
**17**	δ-Cadinene	16.86	1568	2.55	>90
**18**	2,5-Dimethyl-3-vinylhexa-1,4-diene	17.11	1582	0.88	>90
**19**	Didehydro-cycloisolongifolene	17.66	1611	0.29	>90
**20**	Neoisolongifolene,8,9-dehydro	18.05	1631	1.79	>90
**21**	Calamenene cis	18.18	1638	0.28	>90
**22**	1(2*H*)-naphthalenone,7-(1,1-dimethylethyl)-3,4-dihydro-	18.84	1673	0.50	>90
**23**	Decyldiamine	19.36	1701	2.24	>90
**24**	(*E*)-3-Buten-2-one,4-(2,6,6-trimethyl-1-cyclohexen-1-yl)	21.05	1790	0.11	>90
**25**	2-Isopropyl-5-methyl-2-cyclohexen-1-one	21.42	1809	0.59	>90
**26**	5-Amino-2-methoxy-4-picoline	21.97	1838	1.34	>90

**Table 2:** Antioxidant Activity of CAEO and ascorbic acid determined by the DPPH Assay.

Sample	Concentration Range Tested	IC_50_ *	R^2^	Maximum RSA
CAEO	156.25–10,000 µg/mL	3704.83 µg/mL	0.988	62.96%
Ascorbic acid (control)	25–200 µg/mL	58.56 µg/mL	0.98	91.13%

* IC 50 was calculated using a logarithmic trendline.

**Table 3:** Docking scores of CAEO components (∆G kcal/mol); NL-native ligand.

Protein Target (PDB ID)										
Compound	EGFR1 (1XKK)	PDPK1 (2PE1)	BCL-XL (2YXJ)	MEK1 (3DV3)	VEGFR2 (4ASD)	PI3Kγ (4FA6)	AKT (4GV1)	mTOR (4JSX)	BCL-2 (4LVT)	PI3Kα (6GVF)
**NL**	−11.7	−10.1	−10.6	−8.9	−12.1	−8.9	−8.9	−11.2	−11.4	−9.1
**1**	−6.6	−7.3	−7.3	−6.8	−6.2	−6.7	−5.8	−5.5	−6.9	−6.6
**2**	−7.7	−8.3	−6.9	−7.1	−5.7	−7.7	−6.7	−7.3	−6.7	−7.7
**3**	−5.7	−5.9	−6	−6	−6.2	−5.8	−5.7	−5.6	−5.7	−5.7
**4**	−6.5	−6.3	−6.8	−6.4	−6.9	−6.5	−5.8	−5.3	−6.8	−5.3
**5**	−6.8	−6.8	−6.7	−6.1	−7.2	−6.7	−6.7	−6.2	−6.3	−6.9
**6**	−7.2	−7.7	−7.7	−6.8	−5.8	−7.1	−6.5	−6.3	−6.6	−6.6
**7**	−7.8	−8.3	−6.8	−6.8	−6.1	−7.2	−7.2	−7	−7.1	−7.2
**8**	−7.2	−6.7	−6.9	−6.1	−7.9	−7.1	−5.6	−5.9	−6.6	−7.2
**9**	−7.9	−7.2	−6.5	−7.4	−5	−7.3	−6.4	−7.1	−6.7	−7.1
**10**	−7.3	−7.3	−7	−6.5	−7.8	−7.1	−6	−6.2	−6.6	−7.1
**11**	−6.8	−6.9	−7.2	−6.7	−6.2	−6.7	−6.2	−5.5	−6.5	−6.5
**12**	−7.5	−7.1	−7.2	−7.1	−7.8	−7.1	−7.7	−6.4	−6.6	−7.3
**13**	−7.5	−7.1	−7	−6.6	−5.8	−7.6	−6.6	−6.9	−6.8	−7.3
**14**	−6.8	−7.4	−7.1	−6.4	−5.7	−6.8	−6	−6.3	−6.7	−6.6
**15**	−6.6	−6.2	−7.4	−6.9	−4.8	−6.7	−5.8	−5.6	−7.2	−6.6
**16**	−7.4	−7.1	−7	−6.5	−5.1	−6.7	−6	−6.2	−6.5	−7.4
**17**	−5.1	−5.3	−5.4	−5.4	−6.2	−5.5	−5.5	−5.4	−5.2	−5.4
**18**	−6.4	−6.6	−7.1	−6.5	−6.1	−6.3	−5.5	−5.4	−6.8	−6.5
**19**	−7	−6.3	−7.2	−6.4	−5.8	−6.6	−5.6	−5.5	−6.9	−5.7
**20**	−6.7	−6.9	−7	−6.4	−6.6	−6.4	−5.4	−5.6	−6.7	−6.3
**21**	−7.3	−7.4	−7.4	−6.7	−5.9	−7.1	−6.6	−6.8	−7	−7.5
**22**	−8.3	−8	−7	−7.2	−8.4	−7.3	−7.1	−7.9	−6.9	−7.2
**23**	−5	−4.8	−4.9	−5	−5.4	−5	−4.8	−4.7	−4.6	−4.9
**24**	−6.4	−6.9	−6.1	−6	−6.1	−6.2	−6.3	−6.7	−6.1	−6
**25**	−6.1	−5.9	−5.7	−5.9	−6.2	−5.6	−5.8	−5.7	−5.5	−6.1
**26**	−5.3	−5.2	−4.7	−4.9	−5.4	−5.1	−5.2	−4.8	−4.8	−4.9

**Table 4:** Docking scores of CAEO components calculated as a percentage of the NL score for each protein (100%).

Protein Target (PDB ID)										
Compound	EGFR1 (1XKK)	PDPK1 (2PE1)	BCL-XL (2YXJ)	MEK1 (3DV3)	VEGFR2 (4ASD)	PI3Kγ (4FA6)	AKT (4GV1)	mTOR (4JSX)	BCL-2 (4LVT)	PI3Kα (6GVF)
**NL**	100.00	100.00	100.00	100.00	100.00	100.00	100.00	100.00	100.00	100.00
**1**	56.41	72.28	68.87	76.40	51.24	75.28	65.17	49.11	60.53	72.53
**2**	65.81	82.18	65.09	79.78	47.11	86.52	75.28	65.18	58.77	84.62
**3**	48.72	58.42	56.60	67.42	51.24	65.17	64.04	50.00	50.00	62.64
**4**	55.56	62.38	64.15	71.91	57.02	73.03	65.17	47.32	59.65	58.24
**5**	58.12	67.33	63.21	68.54	59.50	75.28	75.28	55.36	55.26	75.82
**6**	61.54	76.24	72.64	76.40	47.93	79.78	73.03	56.25	57.89	72.53
**7**	66.67	82.18	64.15	76.40	50.41	80.90	80.90	62.50	62.28	79.12
**8**	61.54	66.34	65.09	68.54	65.29	79.78	62.92	52.68	57.89	79.12
**9**	67.52	71.29	61.32	83.15	41.32	82.02	71.91	63.39	58.77	78.02
**10**	62.39	72.28	66.04	73.03	64.46	79.78	67.42	55.36	57.89	78.02
**11**	58.12	68.32	67.92	75.28	51.24	75.28	69.66	49.11	57.02	71.43
**12**	64.10	70.30	67.92	79.78	64.46	79.78	86.52	57.14	57.89	80.22
**13**	64.10	70.30	66.04	74.16	47.93	85.39	74.16	61.61	59.65	80.22
**14**	58.12	73.27	66.98	71.91	47.11	76.40	67.42	56.25	58.77	72.53
**15**	56.41	61.39	69.81	77.53	39.67	75.28	65.17	50.00	63.16	72.53
**16**	63.25	70.30	66.04	73.03	42.15	75.28	67.42	55.36	57.02	81.32
**17**	43.59	52.48	50.94	60.67	51.24	61.80	61.80	48.21	45.61	59.34
**18**	54.70	65.35	66.98	73.03	50.41	70.79	61.80	48.21	59.65	71.43
**19**	59.83	62.38	67.92	71.91	47.93	74.16	62.92	49.11	60.53	62.64
**20**	57.26	68.32	66.04	71.91	54.55	71.91	60.67	50.00	58.77	69.23
**21**	62.39	73.27	69.81	75.28	48.76	79.78	74.16	60.71	61.40	82.42
**22**	70.94	79.21	66.04	80.90	69.42	82.02	79.78	70.54	60.53	79.12
**23**	42.74	47.52	46.23	56.18	44.63	56.18	53.93	41.96	40.35	53.85
**24**	54.70	68.32	57.55	67.42	50.41	69.66	70.79	59.82	53.51	65.93
**25**	52.14	58.42	53.77	66.29	51.24	62.92	65.17	50.89	48.25	67.03
**26**	45.30	51.49	44.34	55.06	44.63	57.30	58.43	42.86	42.11	53.85

**Table 5:** Structural characteristics and docking parameters of the protein targets used for the molecular docking of CAEO components, including domain annotations, crystallized residue ranges, and native co-crystallized ligands employed as positive controls.

Protein (PDB ID)	Grid Box Center (x, y, z), (Å)	Grid Box Size (x, y, z), (Å)	Chain/Domain	Residue Range	Native Ligand	Ref.
**PDPK1 (2PE1)**	−4.4286, 44.7066, 34.1136	25.0, 14.7699, 25.0	A/Kinase	82–360	BX-517	[68]
**mTOR (4JSX)**	48.7823, −2.3760, −47.9124	15.0708, 15.0145, 15.0145	A/Kinase (ΔN-mTOR)	2015–2549	Torin2	[69]
**MEK1 (3DV3)**	40.2064, −13.4927, −4.9430	9.6529, 17.1643, 25.0	A/Kinase	59–364	PF04622664	[70]
**AKT/PKB (4GV1)**	−20.6286, 4.1495, 16.2279	15.8042, 9.6797, 25.0	A/Kinase	144–480	AZD5363	[71]
**PI3Kα (6GVF)**	−18.9373, 147.5424, 20.0002	11.5870, 11.9664, 25.0	A/Catalytic p110α	107–1051	Sapanisertib	[72]
**PI3Kγ (4FA6)**	44.0671, 13.4579, 25.7492	14.3086, 12.8763, 25.0	A/Catalytic	144–1102	PI3Kalpha/mTOR-IN-1	[73]
**BCL-2 (4LVT)**	7.5384, −2.9382, −8.2927	14.9275, 29.9577, 16.1318	A/BH3-binding	1–34, 92–207	Navitoclax	[74]
**BCL-XL (2YXJ)**	−9.1464, −13.4285, 1.7349	16.4613, 22.7737, 25.0	A/BH3-binding	1–209	ABT-737	[75]
**EGFR1 (1XKK)**	19.2063, 34.5200, 34.6792	25.0, 11.5551, 24.2206	A/Kinase	695–1028	Lapatinib	[76]
**VEGFR2 (4ASD)**	−23.1483, 0.6107, −3.7034	17.6071, 9.7250, 25.0	A/Kinase	787–1171	Sorafenib	[77]

## Data Availability

The original contributions presented in the study are included in the article; further inquiries can be directed to the corresponding author.

## Supplementary Materials

The following supporting information can be downloaded at: https://www.mdpi.com/article/10.3390/molecules31010046/s1, Table S1: SMILES of PI3Kγ active and decoy ligands used for ROC analysis.
