# Supplementary material for: Evaluation of Cedrus atlantica Essential Oil: Chemical Composition, Anticancer Activity and Molecular Docking Studies
Source: Molecules. 2025 Dec 22;31(1):46. doi: 10.3390/molecules31010046 (PMC12786621; doi:10.3390/molecules31010046)
Supplement: Supplementary file 1 [file molecules-31-00046-s001.zip › molecules-4038019-supplementary.pdf]

Supplementary Materials

Evaluation of *Cedrus atlantica* Essential Oil: Chemical Composition, Anticancer Activity and Molecular Docking Studies

Silvia Gruin [1], Octavian Crețu [1], Alexandra Mioc [2,3] *, Marius Mioc [2,3], Alexandra Prodea [2,3], Elisabeta Atyim [2,3], Alexandra Teodora Lukinich-Gruia [4], Maria-Alexandra Pricop [4,5], Armand Gogulescu [1] and Codruța Șoica [2,3]

Table S1. SMILES of PI3Kγ active and decoy ligands used for ROC analysis

ID	Suplementary identifier	SMILE	PDB ENTRY
Active 1	AS604850	O=C1C(\SC(=O)N1)=C\c3ccc2OC(F)(F)Oc2c3	2A4Z
Active 2	AS605240	O=C1C(\SC(=O)N1)=C\c3cc2nccnc2cc3	2A5U
Active 3	PIK-39	Clc2cccc3N=C(N(c1ccccc1OC)C(=O)c23)CSc5ncnc4c5ncn4	2CHW
Active 4	PIK-90	O=C(c1cccnc1)Nc3nc2c(OC)c(OC)ccc2c4[n+]3ccn4	2CHX
Active 5	CH5132799	O=S(=O)(N3c2nc(nc(c1cnc(nc1)N)c2CC3)N4CCOCC4)C	3APC
Active 6	CH5108134	n1c4c(c(nc1N2CCOCC2)c3cnc(nc3)N)CCN4c5cccnc5	3APD
Active 7	CH5039699	n1c4c(c(nc1N2CCOCC2)c3cccc(O)c3)CCN4c6ccc5ncnc5c6	3APF
Active 8	GDC0941	O=S(=O)(N1CCN(CC1)Cc3sc2c(nc(nc2c3)c5cccc4nncc45)N6CCOCC6)C	3DBS
Active 9	GSK2126458	COc1ncc(cc1N[S](=O)(=O)c2ccc(F)cc2F)c3ccc4nccc(c5ccnnc5)c4c3	3L08
Active 10	PF-04691502	O=C2C(=Cc1c(nc(nc1C)N)N2C3CCC(OCCO)CC3)c4ccc(OC)nc4	3ML9
Active 11	Buparlisib	FC(F)(F)c1cc(ncc1c3nc(nc(N2CCOCC2)c3)N4CCOCC4)N	3SD5
Active 12	Apitolisib	O=C(N1CCN(CC1)Cc3sc2c(nc(nc2c3C)c4cnc(nc4)N)N5CCOCC5)C(O)C	3TL5
Active 13	Bimiralisib	Nc1cc(c(cn1)c2nc(nc(n2)N3CCOCC3)N4CCOCC4)C(F)(F)F	5OQ4
Active 14	Gedatolisib	C(N1CCC(N(C)C)CC1)(c6ccc(NC(Nc2ccc(cc2)c4nc(N3CCOCC3)nc(n4)N5CCOCC5)=O)cc6)=O	7JWE

Active 15	AMG-511	O=S(=O)(N4CCN(C(c2cc(c1nc(nc(n1)N)C)c(nc2)Nc3cc(F)c(OC)nc3)C)CC4)C	4FLH
Active 16	PF-04979064	O=C(N5CCC(N3C(=O)N(c2cnc1ccc(nc1c23)c4ccc(nc4)C)C)CC5)C(O)C	4HVB
Active 17	IPI-549	Cn1cc(cn1)C#Cc6cccc2c6C(=O)N(C(=C2)C(C)NC(=O)c4c3ncccn3nc4N)c5ccccc5	6XRL
Active 18	AZD-7648	CN1C(=O)N(C2CCOCC2)c3nc(Nc4cn5ncnc5cc4C)ncc13	6T3C
Active 19	AZD-6482	O=C(O)c1ccccc1NC(C=4C3=NC(N2CCOCC2)=CC(=O)N3C=C(C=4)C)C	4URK
Active 20	Copanlisib	COc1c(OCCCN2CCOCC2)ccc3C4=NCCN4C(=Nc13)NC(=O)c5cnc(N)nc5	5G2N
Decoy 1	-	COc1ccc(cc1OC)[C@@H]2C(=C3N(C(=O)[C@H](S3)CC(=O)[O-])C(=C2C(=O)OC)N)C#N	-
Decoy 2	-	COc1cc(ccc1O)[C@@H]2C(=C3N(C(=O)[C@@H](S3)CC(=O)[O-])C(=C2C(=O)OC)N)C#N	-
Decoy 3	-	CCOC(=O)C1=C(N2C(=O)[C@@H](SC2=C([C@H]1c3ccc(c(c3)OC)O)C#N)CC(=O)[O-])N	-
Decoy 4	-	CCc1nnc(s1)[N-]S(=O)(=O)c2ccc(cc2)c3ccc(o3)C=C4C(=O)NC(=S)NC4=O	-
Decoy 5	-	CCCCN1[C@H]([C@@H](C(=NC1=O)[O-])N(CCC)C(=O)CCc2c(c(c(=O)[nH]c2C)C#N)C)N	-
Decoy 6	-	CCOC(=O)C1=C(NC(=O)N[C@H]1c2ccc3c(c2)OCO3)CSC4=NC(=O)CC(=N4)[NH-]	-
Decoy 7	-	CCOC(=O)[C@@H]1CCCN(C1)c2[nH]c3c(c(=O)n2)[C@H](C(=C(N3)[O-])C#N)c4ccc(c(c4)OC)O	-
Decoy 8	-	CC1(CC2=C(C(=O)C1)[C@](C(=O)N2Cc3ccco3)(C(F)(F)F)[N-]S(=O)(=O)c4nnc(s4)N)C	-
Decoy 9	-	CCN(C(=O)[C@H](/C=N\NC(=O)CCCNc1c(cc(cn1)C(F)(F)F)Cl)C#N)C(=O)[O-]	-
Decoy 10	-	CCOc1ccc(cc1)S(=O)(=O)Nc2ccc(cc2)C(=O)NNC(=O)c3ccc(nn3)[O-]	-
Decoy 11	-	c1cc(cc(c1)Cl)N2C(=O)C[C@H](C2=O)N(CCc3ccc(cc3)S(=O)(=O)N)C(=O)/C=C\C(=O)[O-]	-
Decoy 12	-	COc1ccc(cc1)S(=O)(=O)Oc2cccc(c2)/C=N\N=C\3/NC(=O)[C@H](S3)CC(=O)[O-]	-

Decoy 13	-	COc1ccc(cc1)S(=O)(=O)Oc2cccc(c2)/C=N/N=C/3\NC(=O)[C@H](S3)CC(=O)[O-]	-
Decoy 14	-	Cc1c(cnn1CCC(=O)N[C@@H]2[C@H]3N(C2=O)C(=C(CS3)CSc4nnc(s4)C)C(=O)[O-])[N+](=O)[O-]	-
Decoy 15	-	c1cc2c(cc1/C=C\3/C(=O)NC(=O)S3)OC(O2)(F)F	-
Decoy 16	-	Cc1c(sc(c1C#N)NC(=O)c2c(nn(c2C)C)C)C	-
Decoy 17	-	c1ccc(c(c1)CN2C(=O)C3(CCCC3)NC2=O)C#N	-
Decoy 18	-	CC1CCC2(CC1)C(=O)N(C(=O)N2)/N=C/c3ccccc3	-
Decoy 19	-	[H]/N=C/1\[C@@H](C2=C(CCCC2)[C@@H](C1(C#N)C#N)c3ccco3)C#N	-
Decoy 20	-	C[C@H]1C[C@@H]1C(=O)Nc2nc-3c(s2)CCc4c3cnn4C	-
Decoy 21	-	c1cc(c(cc1Br)[N+](=O)[O-])N2CCNCC2	-
Decoy 22	-	Cn1cc(c2c1cccc2)c3cc4n(n3)CCC(=O)N4	-
Decoy 23	-	Cc1cc[nH]/c(=N\S(=O)(=O)c2cccnc2Cl)/c1	-
Decoy 24	-	Cn1c(=S)[nH]nc1N2CCN(CC2)c3ccc(cc3)F	-
Decoy 25	-	C[C@H](C12CC3CC(C1)CC(C3)C2)N4C(=O)C(=O)NC4=O	-
Decoy 26	-	Cc1cccc(c1)[C@H]2CC(=O)NC3=NC(=NC(=O)[C@@H]23)C	-
Decoy 27	-	C1CC[C@H]2[C@@H](C1)N(CCO2)C(=O)NC(=O)CCCl	-
Decoy 28	-	Cc1c(cnn1c2ccc(cc2)Cl)c3n[nH]c(=S)o3	-
Decoy 29	-	c1cc2c(cc1F)N(CCC2)S(=O)(=O)c3c[nH]nc3	-
Decoy 30	-	c1cc(=O)[nH]nc1C(=O)N2CC3C[C@H]4C[C@@H](C3)CC2C4	-
Decoy 31	-	C[NH+]1CCC[C@@H](C1)N(C)c2ccc3c(c2)N=C(C3=O)[O-]	-

Decoy 32	-	C[C@@H]1CCCN([C@H]1C)C(=O)N2CCC[C@@H](C2)CC(=O)O	-
Decoy 33	-	C[C@@H]1C2=C(CC(CC2=O)(C)C)Nc3n1nc(n3)SC	-
Decoy 34	-	C1CCCC2(CC1)C(=O)N(C(=O)N2)CN3CCCC3	-
Decoy 35	-	c1ccc(cc1)[C@@H]2C[C@@H](n3cnnc3N2)c4ccco4	-
Decoy 36	-	c1ccc2c(c1)NC(=O)CN2C(=O)c3cccn3C4CC4	-
Decoy 37	-	c1c2c(nc(c1C#N)[NH+]3CC[C@H](C3)C(=O)[O-])CCCC2	-
Decoy 38	-	Cc1c2ccccc2c(nn1)N[C@@H]3CCCS(=O)(=O)C3	-
Decoy 39	-	CC1(CCCN(C1)C(=O)N2CCC[C@H](C2)CC(=O)O)C	-
Decoy 40	-	c1cc(ccc1CN2C(=O)NC(=O)C23CCCCC3)C#N	-
Decoy 41	-	c1ccc2c(c1)[C@@]3(CCCN(C3)c4cccc(n4)C#N)C(=O)N2	-
Decoy 42	-	Cc1ccc(cc1)n2ccnc2c3cc4n(n3)CCCNC4	-
Decoy 43	-	c1cc(c(c(c1)Cl)O)/C=N/C23CN4CN(C2)CN(C3)C4	-
Decoy 44	-	C[C@H]1CCCN(C1)C(=O)C2CCN(CC2)C(=O)NCC#C	-
Decoy 45	-	c1cc2c(cc1[C@@H]3[C@@H](CCCO3)C(=O)O)OCCCO2	-
Decoy 46	-	CC1(CCCN1S(=O)(=O)c2cc(c(=O)[nH]c2)Cl)C	-
Decoy 47	-	c1c(sc(n1)NC(=O)c2csnn2)Br	-
Decoy 48	-	CCc1c(c(=O)nc([nH]1)c2cnn(c2)C)Br	-
Decoy 49	-	CC1(C(=O)N(C2(CCCCC2)C(=O)N1)C[C@@H]3CCOC3)C	-
Decoy 50	-	C1CCC2(CC1)C(=O)N(CC(=O)N2)C[C@H]3CCCOC3	-
Decoy 51	-	C[C@@H]1C[C@H](CN(C1)S(=O)(=O)N2C[C@@H](NC[C@@H]2C)C)C	-
Decoy 52	-	C[C@@H]1CN[C@@H](CN1c2c3ccccc3nnc2C#N)C	-

Decoy 53	-	c1ccc2c(c1)C[C@@H]2c3nc(on3)c4c(ccc[nH+]4)[O-]	-
Decoy 54	-	CCc1c(c(nc(n1)c2c[nH]nn2)Cl)Br	-
Decoy 55	-	c1cc2c(cc1C3(CCC4(CC3)OCCO4)O)OCCO2	-
Decoy 56	-	CCNC(=O)N1CCN(CC1)C2C[C@H](O[C@@H](C2)C)C	-
Decoy 57	-	c1ccc2c(c1)C[C@H](O2)CN3C(=O)NC(=O)C34CCCC4	-
Decoy 58	-	CN1CCCN(CC1)c2cc3c(cc2Cl)C(=O)C(=O)N3	-
Decoy 59	-	C[C@H]1CCCN1C(=O)N2[C@@H]3CC[C@H]2CC(C3)CC(=O)O	-
Decoy 60	-	C[C@@H]1[C@H](CC[NH+]1c2c3c(ncn2)CCCCC3)C(=O)[O-]	-
Decoy 61	-	Cn1ccc(n1)n2cncc2c3cccc4c3NCCC4	-
Decoy 62	-	Cc1nnc2n1C[C@H](CC2)Nc3nc4ccccc4s3	-
Decoy 63	-	CC[C@H]1CC[C@@H]([C@@H](C1)N2CC(=O)NC(=O)C2(C)C)C#N	-
Decoy 64	-	C[C@@H]1CCC[C@@H](N1C(=O)C[C@@H]2CS(=O)(=O)CCN2)C	-
Decoy 65	-	C1Cc2c3c(nc4n(c3=O)CCC4)sc2/C(=N\O)/C1	-
Decoy 66	-	COc1ccc2c(c1OC)nc(n3c2ncc3)NC(=O)c4cccnc4	-
Decoy 67	-	Cc1c(c(n(n1)c2ccccc2)C)/C=N/Nc3ccc4nncn4n3	-
Decoy 68	-	c1ccc(cc1)N2C(=O)[C@@H](C(=O)NC2=O)/C=N/Cc3ccc4c(c3)OCO4	-
Decoy 69	-	c1cc(c(cc1Cl)c2nnc(o2)NC(=O)CN3C(=O)CCC3=O)Cl	-
Decoy 70	-	C[C@@]1(C(=O)N(C(=O)N1)CC(=O)N2CCCCC2)c3ccc4c(c3)OCO4	-
Decoy 71	-	c1cc2c(cc1[N+](=O)[O-])sc(n2)NC(=O)C[C@@H]3CCS(=O)(=O)C3	-
Decoy 72	-	c1cc2c3cc(ccc3oc2cc1n4cnnn4)S(=O)(=O)NC5CC5	-

Decoy 73	-	COc1cc(c(c(c1)OC)[C@@H]2NS(=O)(=O)N=C3N2CCC3)OC	-
Decoy 74	-	c1cc(c(cc1S(=O)(=O)N2CCCC2)[N+](=O)[O-])N3CCC[C@H](C3)O	-
Decoy 75	-	CC(=O)NC1=NN([C@H](S1)/C=C/c2ccccc2[N+](=O)[O-])C(=O)C	-
Decoy 76	-	Cc1c(c(=O)n2ccsc2n1)NC(=O)COc3ccc4c(c3)OCO4	-
Decoy 77	-	Cc1cc(no1)NC(=O)[C@@H](c2ccccc2)Sc3nnnn3C	-
Decoy 78	-	Cc1c(sc(c1C#N)NC(=O)Cn2c(c(c(n2)C)[N+](=O)[O-])C)C	-
Decoy 79	-	CCn1c(ccn1)[C@H]2C(=C(Nc3n2nc(n3)SC)C)C(=O)OC	-
Decoy 80	-	Cc1c(c(n(n1)CC(=O)Nc2ccc(cn2)Br)C)[N+](=O)[O-]	-
Decoy 81	-	Cc1cc(nc(n1)SCC(=O)Nc2cccc(c2)n3cnnn3)C	-
Decoy 82	-	Cn1cc(cc1C(=O)NCCc2ccc3c(c2)OCCO3)[N+](=O)[O-]	-
Decoy 83	-	Cc1cc(n2c(n1)nc(n2)CC(=O)NCc3ccc4c(c3)OCO4)C	-
Decoy 84	-	Cn1c2ccc(cc2nn1)C(=O)N(C)CC(=O)Nc3cccc(c3)C#N	-
Decoy 85	-	CN1CC(=O)N(c2ccccc2C1=O)CC(=O)Nc3ccc4c(c3)OCO4	-
Decoy 86	-	COC(=O)[C@H]1C[C@]2(c3cc(ccc3NC2=O)[N+](=O)[O-])N4[C@@H]1CCC4	-
Decoy 87	-	Cc1cccc(c1)n2c(n[nH]c2=S)Cn3c(c(c(n3)C)[N+](=O)[O-])C	-
Decoy 88	-	c1ccnc(c1)/C=N/NC(=O)[C@H]2C=C(N=N2)c3ccc4c(c3)OCO4	-
Decoy 89	-	Cc1ccc(cc1)n2cc(nn2)C(=O)NCc3nnc4n3CCCCC4	-
Decoy 90	-	Cc1cc(on1)NC(=O)[C@@H](C)Sc2nc3nc(c(c(n3n2)C)C)C	-
Decoy 91	-	Cn1c2c(=O)[nH]c(=O)n(c2nc1c3csnn3)Cc4ccccc4	-
Decoy 92	-	Cn1c(=O)c2ccccc2n(c1=O)CC(=O)NCc3ccc4c(c3)OCO4	-
Decoy 93	-	Cc1cc(on1)CN2C(=O)[C@](NC2=O)(C)c3ccc4c(c3)OCCCO4	-

Decoy 94	-	c1cc(=O)n(cc1[N+](=O)[O-])Cc2[nH]c(=O)c3c4c(sc3n2)CCC4	-
Decoy 95	-	c1ccc(c(c1)N2C(=O)/C(=C/c3ccc(o3)[N+](=O)[O-])/C(=O)NC2=S)F	-
Decoy 96	-	Cc1nc(c(n1CC(=O)NC2(CCCC2)C#N)Br)[N+](=O)[O-]	-
Decoy 97	-	c1ccc(c(c1)CN2C(=O)c3ccccc3S(=O)(=O)N2)[N+](=O)[O-]	-
Decoy 98	-	Cc1ccc(c(c1C)n2cnnn2)C(=O)Nc3nnc(s3)C4CC4	-
Decoy 99	-	CCc1nc(no1)CN2CCC[C@H](C2)N(C)c3c4cc[nH]c4ncn3	-
Decoy 100	-	Cc1c2cc(cnc2n(n1)C)S(=O)(=O)Nc3ccc4c(c3)OCO4	-
